# Elevated remnant cholesterol impairs sleep characteristics in individuals with newly diagnosed moderate-to-severe obstructive sleep apnea: a cross-sectional case–control study

**DOI:** 10.3389/fneur.2025.1648807

**Published:** 2025-09-10

**Authors:** Yanling Liu, Fengying Zhang, Ping Zhang, Li Chen

**Affiliations:** The Affiliated Nanhua Hospital, Department of Neurology, Hengyang Medical School, University of South China, Hengyang, China

**Keywords:** obstructive sleep apnea, remnant cholesterol, apnea-hypopnea index, oxygen desaturation index, polysomnography

## Abstract

**Background:**

Although obstructive sleep apnea (OSA) is widely recognized as a common contributor to disruptions in sleep and lipid metabolism, there is still a lack of concrete evidence to substantiate this correlation. Remnant cholesterol (RC) is increasingly being acknowledged as a lipid-related risk factor for many diseases; however, its role in sleep remains uncertain. We aimed to assess whether RC is associated with OSA disease events and to determine the impact of RC on sleep parameters.

**Methods:**

This cross-sectional case–control study recruited141patients recently diagnosed with moderate-to-severe OSA (based on overnight polysomnography) and 47 healthy participants who served as controls. We performed a PSG assessment and collected sleep parameters and biochemical, demographic, and clinical data.

**Results:**

Compared to controls (19.1%), patients with OSA exhibited a significantly higher prevalence of elevated RC (36.1%). Patients with OSA had higher serum RC levels than controls [0.37 (0.19–0.56) vs. 0.45 (0.255–0.78) mmol/L; *p* = 0.013]. Compared to patients with OSA without elevated serum RC, those with high RC levels exhibited statistically significant differences in wake frequency; the percentage time in non-rapid eye movement stage 1 (NREM1, N1), N2%, and N3 sleep; Apnea-Hypopnea Index (AHI); Oxygen Desaturation Index (ODI); Breathing-related arousal index. (BAI); and average SpO2. Pearson correlation analysis showed that the serum RC concentration in patients with OSA was positively correlated with wake frequency, percentage time in N1, AHI, ODI, and BAI, whereas it was negatively correlated with percentage time in N2 and N3 and mean nocturnal oxygen saturation.

**Conclusion:**

For the first time, we found that elevated RC levels are highly prevalent and significantly associated with impaired sleep architecture and respiratory parameters in patients with newly diagnosed OSA. Therefore, serum RC assessment should be included in the work-up for the diagnosis of OSA.

## Introduction

Obstructive sleep apnea (OSA) is characterized by recurring apneic events that lead to hypoxia, hypercapnia, and sleep disruption (1). This condition, the most common form of sleep-disordered breathing, is associated with an increased risk of cardiovascular complications, including hypertension, stroke, heart failure and sudden cardiac death (2). However, many patients fail to receive timely and effective treatments, leading to poor outcomes. Poor sleep quality is frequently attributed to OSA and indicates a poor quality of life in patients (3). Understanding poor sleep quality during the early stages of the disease is important because sleep quality improves with OSA treatment, which may mask the prognosis of OSA. To improve interventions, there is an urgent need to understand specific sleep-related parameters in patients with OSA, especially during the early stages of the disease.

Dyslipidemia is an intermediary exacerbating factor in various diseases, and its role in OSA has drawn increasing attention in recent years. Clinical and epidemiological findings progressively suggest that the prevalence of dyslipidemia in individuals with OSA escalates as OSA becomes more severe (4–6). Notably, OSA is independently associated with the dysregulation of lipoprotein metabolism (7). Intermittent hypoxia and sleep fragmentation, which are fundamental pathophysiological aspects of OSA, play crucial roles in dyslipidemia, resulting in elevated sympathetic activity, increased oxidative stress, and systemic inflammation (8, 9). In patients with OSA, combined adverse lipid profiles are more likely to result in poor prognoses (10). Hence, screening and early detection of sleep parameters in patients with OSA-associated dyslipidemia are critical for improving outcomes. However, there are few studies on this topic and specific effective clinical indicators for these patients.

Remnant cholesterol (RC), also known as triglyceride-rich lipoprotein cholesterol, is an emerging biomarker involved in multiple conditions, including both metabolic disorders and cardiovascular (11). Previous research on cholesterol metabolism has predominantly focused on LDL-C and HDL-C (12, 13). Notably, new findings suggest that, unlike LDL-C, RC is taken up by macrophages without needing oxidative modifications (14), making it as or possibly more atherogenic than LDL-C (15). In addition, RC, rather than triglycerides (TG), was reported to increase the risk of atherosclerotic cardiovascular disease (16). These results suggest that RC could potentially complement and even have unique advantages over the currently available biomarkers in predicting disease outcomes and thus may guide personalized treatment plans. Evidence has shown that sleep disorders can affect the concentration of lipid profiles in the plasma, such as TG, cholesterol, and lipoproteins; however, the role of the RC in OSA remains unclear.

This study, for the first time, aimed to assess the prevalence of elevated RC in patients newly diagnosed with OSA and to analyze the relationships between serum RC levels and polysomnographic (PSG) parameters in the early phase of the disease.

## Methods

### Study design

**Figure 1:**
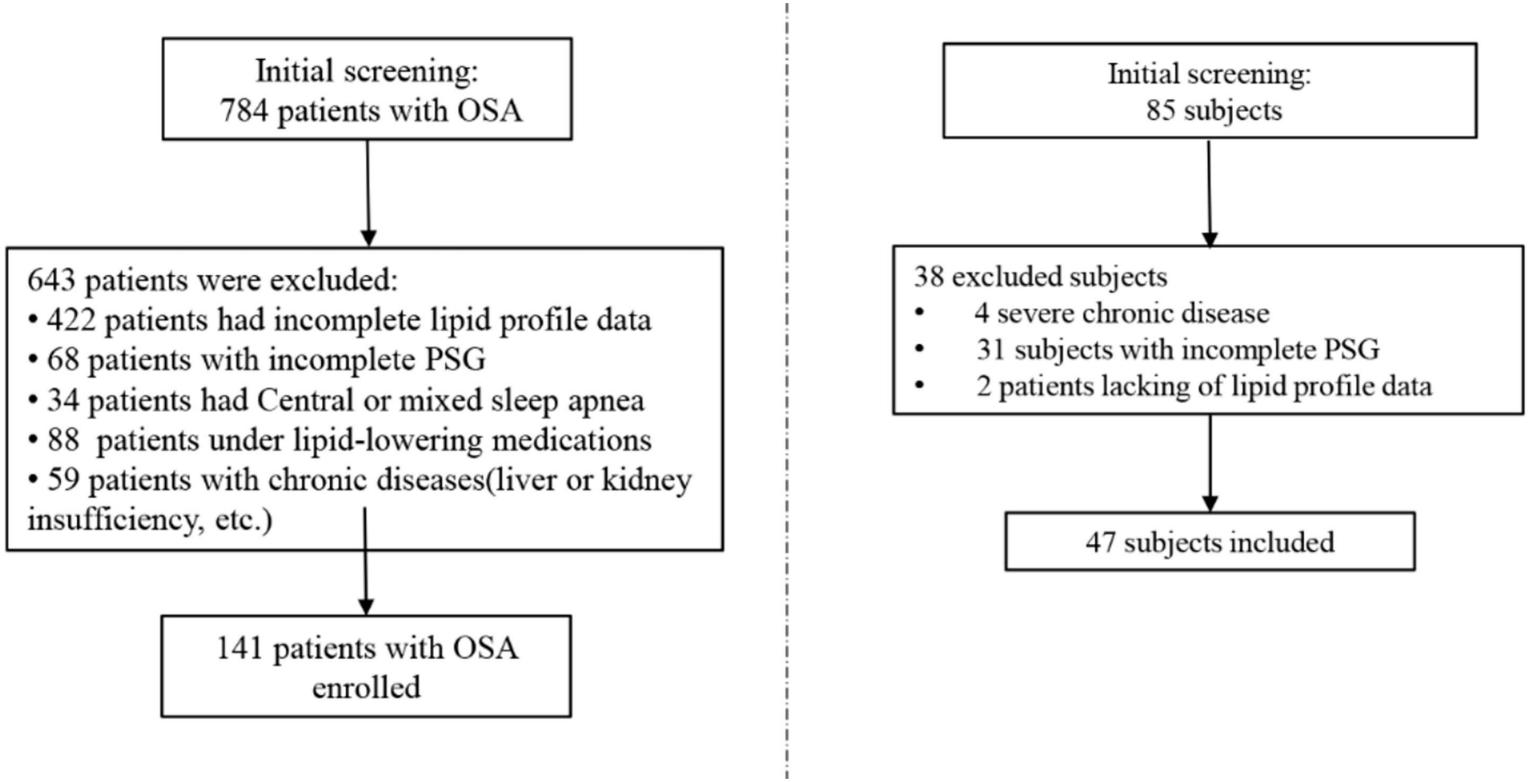
Flowchart illustrating the screening process used for selecting eligible participants.

A total of 784 patients, first diagnosed with OSA at Nanhua Hospital, affiliated with the University of South China, were reviewed in this study, covering the period from January 1, 2023, to June 1, 2024. The exclusion criteria were defined as: (1) age less than 18 or greater than 80 years; (2) other sleep disorders (mixed sleep apnea, narcolepsy, idiopathic hypersomnia, restless legs syndrome etc.), medications impacting sleep/body weight, or substantial; (3) malignancies, infectious diseases, severe hepatic or renal insufficiency, or notable comorbidities; (4) incomplete RC, BMI, or PSG data. The final cohort comprised 141 participants in the OSA group and 47 in the non-OSA group (Figure 1).

To provide a true OSA-free baseline, minimize selection bias, and ensure that observed differences in metabolic parameters are attributable to OSA itself rather than other confounding sleep conditions, we selected healthy controls without OSA as the control group. Inclusion criteria:(1) Age between 18 and 80 years; (2) No symptoms or history of sleep disorders; (3) AHI < 5 events/h as confirmed by overnight PSG; (4) No use of medications affecting sleep or body weight; (5) Willingness to participate and provide informed consent; (6) Complete demographic, anthropometric, and PSG data available.

### Data source and collection

Data on patients were compiled from the electronic medical records system, including key demographic details, clinical history, blood analysis results, and significant medical imaging findings. The blood specimens for analysis were collected in the morning via routine fasting venous blood draws by skilled medical professionals. RC was derived as follows: RC = total cholesterol (mmol/L)—HDL-C (mmol/L)—LDL-C(mmol/L). Fasting remnant cholesterol ≥0.8 mmol/L can be considered as high RC levels (16).

### Polysomnography

All participants underwent overnight polysomnography (PSG), which was analyzed using the Nox A1 Noxturnal system (Nox Medical, Reykjavik, Iceland). The recorded data was divided into 30-s epochs and scored according to the sleep scoring criteria outlined in the AASM Manual for the Scoring of Sleep and Associated Events, v. 2.6.36 (17). Various physiological signals were recorded during the study by using numerous techniques and devices, including electroencephalography (EEG) to measure brain activity, electrooculography (EOG) to detect eye movements, electromyography (EMG) to assess muscular tension (recorded from the chin and tibial electrodes), a nasal pressure sensor to measure airflow, and inductive plethysmography to evaluate chest and abdomen movements. In addition to these recorded signals, the patient’s body position and bilateral masseter EMG were also captured. Episodes of bruxism were classified according to the AASM standards, distinguishing between phasic, tonic and mixed forms. Arousals were categorized as spontaneous, respiratory, bruxism, and periodic limb movement (PLM) arousals. All PSG recordings were scored and interpreted by two experienced sleep specialists, both of whom were blinded to the participants’ clinical and radiological data.

### Statistical analysis

Presented data include mean ± standard deviation (SD), medians (interquartile range), or numbers (percentage), depending on their normal distribution, skewness, or categorization. Differences in baseline characteristics between the non-OSA and OSA groups were evaluated using the Mann–Whitney U test, one-way ANOVA, or the χ^2^ test, depending on how the data were distributed. The correlation between RC and PSG parameters was assessed using Spearman correlation analysis.

All statistical analyses utilized the SPSS (version 26.0), and *p*-values < 0.05 (two-sided) were deemed statistically significant.

## Results

### Control group vs. OSA group

Table 1 displays the anthropometric, demographic, clinical, and sleep-related features of the participants. We enrolled 141 patients with OSA (88 males/53 females; mean age: 60.30 ± 12.15 years) and 47 control individuals (18 males/29 females; mean age: 55.10 ± 12.47 years) who met the inclusion criteria. Compared to subjects without OSA, those with OSA were older, predominantly male, and exhibited higher adiposity parameters as well as a greater prevalence of hypertension. Patients with OSA had higher Creatinine 74.9 (66.5–89) vs. 66.2 (58.4–78) mmol/L; *p* = 0.022, and T4 16.7 (14.95–18.18) vs. 15.7(14.1–16.93; *p* = 0.033). Conversely, patients with OSA had lower serum HDL-C levels: 1.11 (0.9–1.31) vs. 1.33 (0.95–1.58); *p* = 0.018. As expected, significant differences in multiple sleep parameters were noted between the two groups including N1, N2, N3, AHI, ODI, BAI and average SpO2 (all *P* < 0.05).

**Table 1:** Characteristics of the control group and OSA groups.

	Control group (*n* = 47)	OSA group (*n* = 141)	*p*
Age, y	55.10 ± 12.47	60.30 ± 12.15	**0.019**
Sex, male/female	18/29	88/53	**<0.001**
BMI, kg/m2	22.43(20.55–24.4)	25.2(22.45–28.45)	**<0.001**
SBP, mmHg	126(113–135)	133(121.5–145)	**0.021**
DBP, mmHg	81(72–89)	86(75.5–91)	0.087
Hypertension (%)	5	29	**0.007**
Diabetes (%)	3	13	0.182
Coronary heart disease (%)	2	9	0.260
Smoke use (%)	15	23	0.367
Alcohol use (%)	10	12	0.199
FBS, mmol/l	6.2(5.1–8.23)	6.22(5.2–8.55)	0.529
Creatinine, mg/dL	66.2(58.4–78)	74.9(66.5–89)	**0.022**
UC, mg/dL	2.81 ± 0.90	2.68 ± 0.82	0.372
TG, mmol/l	4.52 ± 0.99	4.43 ± 1.0	0.636
TC, mmol/l	1.37(1.01–2.49)	1.64(1.18–2.46)	0.177
HDL-C, mmol/l	1.33(0.95–1.58)	1.11(0.9–1.31)	**0.018**
LDL-C, mmol/l	310.97 ± 105.11	327.48 ± 97.72	0.401
RC, mmol/l	0.37(0.19–0.56)	0.45(0.255–0.78)	**0.013**
HbA1c, %	5.7(5.3–5.9)	5.8(5.4–6.2)	0.240
T3, nmol/L	4.4(4.14–5.22)	4.57(4.11–5.35)	0.656
T4, nmol/L	15.7(14.1–16.93)	16.7(14.95–18.18)	**0.033**
TSH, mIU/L	1.67(1.43–2.42)	1.59(1.18–2.57)	0.519
Total sleep time, min	392(315.5–444.5)	337.5(273.5–415.75)	**0.041**
Sleep efficiency, %	75(63.0–81.9)	70.5(57.7–79.5)	0.217
Wake time after falling asleep, min	119(72–177)	132.5(83.5–196.25)	0.319
Wakefulness frequency, min	27(19–37)	32(21.5–50.5)	0.068
N1, %	20.3(15.9–34.3)	32.8(24.1–48.15)	**<0.001**
N2, %	46.55 ± 11.37	37.7 ± 15.24	**<0.001**
N3, %	9.9(6.3–16.7)	5.0(0–11.8)	**<0.001**
REM, %	19.9(13.1–23.5)	19.0(13.4–21.4)	0.289
AHI (events/h)	3.7(2.5–4.98)	28.9(22.3–47.05)	**<0.001**
ODI (events/h)	3.2(1.0–4.6)	19.1(9.55–34.9)	**<0.001**
BAI (events/h)	7.8(5.5–11)	28.5(22.4–47.05)	**<0.001**
Average SpO_2_ (%)	89(83–91)	82(71–88)	**<0.001**
Mean pulse, beats/min	61(56–68)	64(58–71)	0.229

Data are expressed as numbers (%), means ± standard deviations, or median (range). AHI, Apnea-hypopnea index; FBS, fasting blood sugar; ODI, Oxygen Desaturation Index; T3, T4: total thyroxine 3; BAI, Breathing-related arousal index. Statistics with significant differences are indicated in bold font.

Elevated RC was detected in 51 (36.1%) patients with OSA and 9 (19.1%) in the control group, showing a significantly higher prevalence among OSA patients (*p* < 0.001). Furthermore, patients with OSA showed significantly higher RC concentrations at admission [0.37 (0.19–0.56) vs. 0.45 (0.255–0.78) mmol/L; *p* = 0.013], than those without OSA.

### OSA group

We identified 51 high remnant cholesterol (HRC) and 90 non-HRC patients with OSA. The clinical and biochemical parameters are detailed in Table 2. Compared with non-HRC subjects, those with HRC were older, mostly female, and had elevated BMI and diastolic blood pressure. Regarding the lipid profile, HRC participants had higher TG and TC levels and lower HDL-C.

**Table 2:** General characteristics and sleep parameters of OSA patients classified by RC concentrations (HRC OSA: RC ≥ 0.8 mmol/L; non-HRC OSA: RC < 0.8 mmol/L).

	HRC OSA (*n* = 51)	non-HRC OSA (*n* = 90)	*p*
Age, y	55.59 ± 15.27	59.67 ± 11.79	0.079
Sex, male/female	38/13	44/46	**0.02**
BMI, kg/m2	25.71(23.53, 29.7)	22.8(20.67, 26.1)	**<0.001**
SBP, mmHg	133.00(123.0, 142.0)	127.0(118.0, 144.0)	0.213
DBP, mmHg	88.0(81.0, 93.0)	81.0(72.0, 88.5)	**0.004**
Hypertension (%)	14	20	0.576
Diabetes (%)	8	8	0.258
Coronary heart disease (%)	5	6	0.552
Smoke use (%)	12	26	0.406
Alcohol use (%)	6	16	0.297
FBS, mmol/l	6.90(5.34, 8.70)	6.00(5.10, 7.75)	0.206
Creatinine, mg/dL	77.0(65.75, 92.75)	71.05(59.0, 81.53)	**0.015**
UC, mg/dL	347.79 ± 91.69	306.67 ± 102.35	**0.008**
TG, mmol/l	2.34(1.37, 375)	1.29(1.02, 1.86)	**<0.001**
TC, mmol/l	4.98 ± 0.89	4.31 ± 0.97	**<0.001**
HDL-C, mmol/l	1.01(0.88, 1.10)	1.25(1.02, 1.54)	**<0.001**
LDL-C, mmol/l	2.87 ± 0.78	2.67 ± 0.86	0.175
HbA1c, %	5.80(5.50, 6.20)	5.70(5.30, 6.00)	0.108
T3, nmol/L	4.80(4.20, 5.37)	4.43(4.13, 5.26)	0.183
T4, nmol/L	16.75(14.25, 17.77)	16.35(14.92, 17.38)	0.962
TSH, mIU/L	1.59(1.38, 2.72)	1.63(1.30, 2.42)	0.696
Total sleep time, m	339.5(289.0, 435.88)	358.0(285.13, 428.38)	0.921
Sleep efficiency, %	69.4(55.1, 83.28)	72.15(60.43, 78.8)	0.942
Wake time after falling asleep, min	138.35(66.13, 199.13)	127.25(85.25, 177.38)	0.997
Wakefulness frequency, min	38.5(22.5, 58.25)	28.0(19.25, 38.75)	**0.006**
N1, %	38.5(25.90, 51.6)	25.40(18.15, 31.70)	**<0.001**
N2, %	43.27 ± 11.68	35.53 ± 16.29	**0.007**
N3, %	3.8(0.0, 9.8)	9.0(5.35, 16.05)	**<0.001**
R, %	18.4(12.08, 22.63)	19.4(13.63, 22.23)	**<0.001**
AHI (events/h)	30.1(19.5, 55.60)	18.3(8.30, 27.05)	**<0.001**
ODI (events/h)	23.3(7.90, 48.70)	5.70(2.15, 16.40)	**<0.001**
BAI (events/h)	20.5(9.40, 40.90)	10.50(4.95, 16.25)	**<0.001**
Average SpO2 (%)	95.0(93.0, 96.0)	96.0(95.0, 97.0)	**0.006**

Data are expressed as numbers (%), means ± standard deviations, or median (range). FBS, fasting blood sugar; T3, T4: total thyroxine 3; AHI, Apnea-hypopnea index; ODI, Oxygen Desaturation Index; BAI, Breathing-related arousal index. Statistics with significant differences are indicated in bold font.

The PSG parameters were compared between the non-HRC and HRC groups. The OSA with HRC group showed a higher wakefulness frequency [38.5(22.5, 58.25) vs. 28.0(19.25, 38.75); *p* < 0.001] after sleep onset than the OSA with HRC group. Furthermore, the HRC group showed a greater percentage of time in N1sleep [38.5 (25.90–51.6) vs. 25.40 (18.15–31.70) %] and percentage of time in N2 sleep (43.27 ± 11.68 vs. 35.53 ± 16.29), and lower N3 sleep 3.8[0.0, 9.8) vs. 9.0(5.35, 16.05); all *p* < 0.001]. Respiratory parameters, including the AHI [30.1 (19.5–55.60) vs. 18.3 (8.30–27.05)], ODI [23.3 (7.90, 48.70) vs. 5.70(2.15, 16.40)], BAI [20.5(9.40, 40.90) vs. 10.50(4.95, 16.25)], and average SpO2, differed significantly between the two groups as expected; however, no significant differences were observed in total sleep time, wake time after falling asleep or sleep efficiency between the groups (all *p* < 0.05).

**Figure 2:**
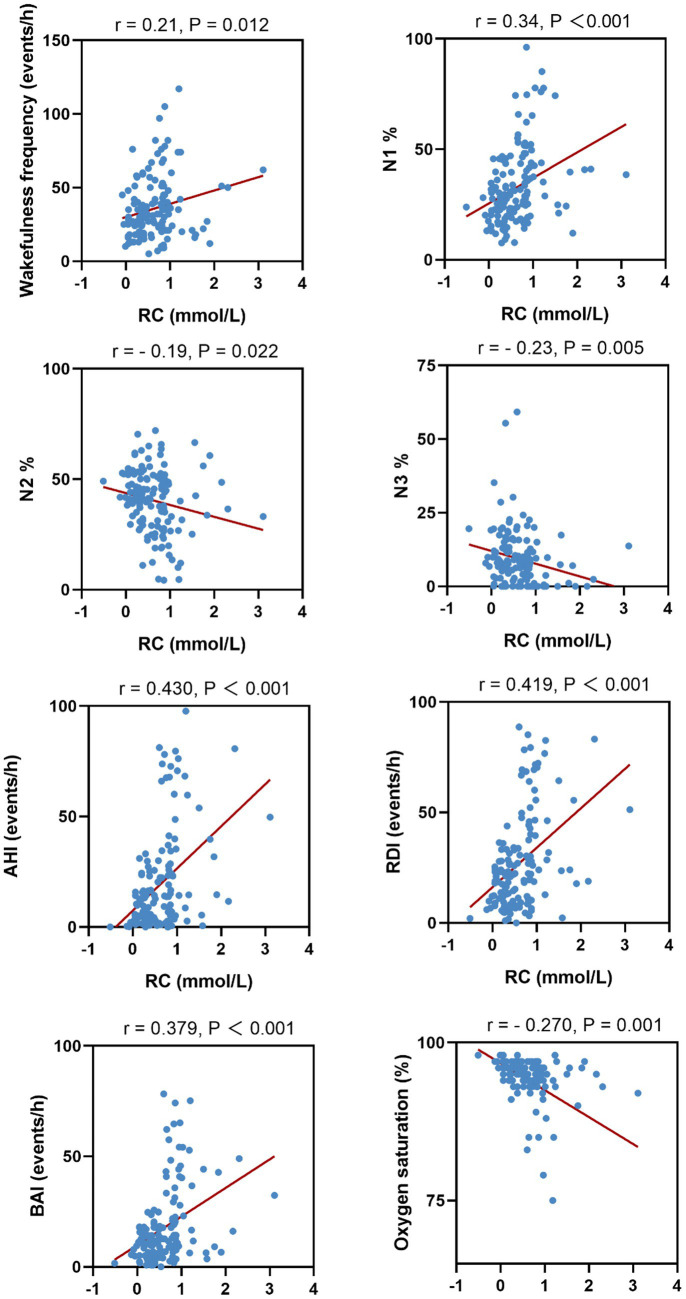
The scatter plot between serum RC concentrations and sleep parameters.

We further analyzed the correlation between serum RC and PSG parameters. Serum RC levels were positively correlated with the wake frequency, percentage time in N1, AHI, ODI, and BAI, whereas it was negatively correlated with percentage time in N2 and N3 and mean nocturnal oxygen saturation. Scatter plots of the four PSG parameters and their correlated features are shown in Figure 2.

## Discussion

This cross-sectional case–control study is the first to report the high prevalence of elevated RC concentrations in a large cohort of patients recently diagnosed with OSA. This study demonstrated an association between RC and OSA. Notably, we also found that RC levels were related to several parameters of sleep fragmentation and structure. Thus, these findings demonstrate a significant association between elevated RC and impaired sleep characteristics.

OSA is connected to changes in lipid metabolism, resulting in higher circulating lipid levels. The altered lipid profiles in OSA are driven by intermittent hypoxia, oxidative stress, and inflammatory mechanisms (18). Although lipid disorders are common in patients with OSA, the lipid profile of this group has recently attracted the attention of sleep physicians. However, findings on the relationship between lipid composition and the OSA state have not been entirely consistent. For instance, a retrospective study involving 2,361 individuals showed that those with OSA had higher TG, increased non-HDL-C, and decreased HDL-C compared to controls (19). In contrast, a prospective cohort study suggested a strong association between HDL-C and severe OSA, while LDL-C was not independently linked (20). Thus, the emergence of conflicting results from different studies highlights the necessity for additional investigation.

RC, a novel biomarker representing cholesterol esters in TG-rich lipoproteins, was calculated as TC minus HDL-C; LDL-C was directly measured. This study validates the high prevalence of elevated RC concentrations in a substantial cohort of patients newly diagnosed with OSA. These findings indicate that combined lipid parameters, particularly RC, may have a stronger correlation with OSA than traditional individual lipid parameters. These findings confirmed the complex interplay between lipid metabolism and OSA pathogenesis. Importantly, these findings add to the growing body of research connecting lipid abnormalities, especially RC, to the risk of OSA.

The high incidence of RC suggests that more clinical attention should be paid to its effects on sleep during the early stages of the disease. Despite many studies confirming the association between sleep disorders and lipid homeostasis in OSA, the results have been varied when it comes to specific lipid indexes. TC, LDL-C, TG, and apolipoprotein B levels have been reported to be higher in patients with OSA than in controls (21). In addition, an independent link was found between AHI changes, HDL-C, and TG levels (22). Among working-age males in Japan, TC levels were associated with short sleep duration, and TG levels were positively associated with the respiratory disturbance index (23). After controlling for confounding factors, higher TG and lower HDL-C levels were associated with nocturnal idiopathic hypersomnia and OSA severity (24). This study suggests that employing a composite index derived from multiple traditional lipids could address the discordance in evaluating different lipid parameters. Our comparative analysis showed that patients with high RC had significantly increased wakefulness frequency, elevated N1 sleep percentage, higher N2 sleep percentage, and reduced N3 sleep stage. However, correlation analysis revealed a relatively weak negative association between RC levels and N2 percentage. An imbalanced distribution of N1, N2, and N3 stages suggests fragmented and insufficient restorative sleep, which is of particular concern because N2 and N3 play critical roles in cognitive and physiological recovery. In particular, a reduction in N3 sleep has been associated with negative health outcomes, such as impaired memory consolidation and increased cardiovascular risk, thereby highlighting the potential adverse effects of disrupted sleep architecture.

Our results revealed, for the first time, the role of RC in sleep parameters. Moreover, considering that OSA, metabolic disorders, and cardiovascular morbidities interact with each other over time (i.e., reciprocal causation), our findings emphasize the importance of early detection and close monitoring of RC in patients with OSA. Notably, RC can be derived from routine, easily accessible blood tests, which are both cost-effective and easily accessible.

The mechanism behind the RC-OSA association has not been fully clarified; we hypothesize that key components of RC, particularly very-LDL-C and IDL-C, are closely linked to the pro-inflammatory activity of macrophages in visceral adipose tissue, which enhances local inflammation through cytokine and chemokine secretion, as well as the production of reactive oxygen and nitrogen species (25). Indeed, compared to traditional lipids parameters, RC shows a stronger correlation with arterial stiffness (26). As expected, in our study, a positive correlation was found between the RC level and several respiratory parameters, including AHI, ODI, and BAI. This suggests that HRC may be associated with a poor prognosis in patients with OSA.

Previous studies have demonstrated that elevated RC is closely associated with ectopic fat deposition, particularly visceral adiposity, which can induce anatomical and functional changes—such as reduced airway caliber and decreased chest compliance—that directly promote upper airway collapsibility and nocturnal hypoxemia, central mechanisms in OSA pathogenesis. Additionally, RC not only serves as a blood lipid marker but also reflects overall metabolic health and is independently linked to adverse obesity phenotypes and related complications. Thus, elevated RC may contribute to the development or progression of OSA through its effects on ectopic fat accumulation and metabolic dysfunction. However, given the complex and potentially bidirectional relationship between RC and sleep fragmentation, and the cross-sectional nature of our study, causality cannot be established. Future longitudinal and mechanistic studies are needed to clarify these associations.

However, this study has several limitations. First, as a cross-sectional study, we only examined correlations between RC levels and sleep parameters, without establishing independent associations or causality. Further prospective studies are needed to validate these associations. Second, key components major RC constituents (very-LDL-C, IDL-C, and chylomicron particles) were not analyzed. Third, the relatively small sample size of the control group may have influenced the findings regarding remnant cholesterol and sleep parameters in the non-OSA population. Finally, the exact time interval between symptom onset and clinical diagnosis of OSA was not systematically collected. Therefore, a recent diagnosis does not necessarily indicate early-stage disease, as there may have been delays in diagnosis.

## Conclusion

To conclude, this research is the first to identify a high prevalence of HRC in a substantial cohort of patients recently diagnosed with OSA, and to establish an association between RC levels and poor sleep architecture and respiratory parameters. Our data suggest that dyslipidemia marked by elevated serum RC levels may be an early event in the development of OSA. We recommend including serum RC assessment in the initial diagnostic process for OSA. By highlighting the role of RC in OSA, this study opens the door to enhanced risk assessment, targeted interventions, individualized management strategies, and preventive.

## Data Availability

The raw data supporting the conclusions of this article will be made available by the authors, without undue reservation.
